# Effect of interleukin-1 blockade with anakinra on leukocyte count in patients with ST-segment elevation acute myocardial infarction

**DOI:** 10.1038/s41598-022-05374-w

**Published:** 2022-01-24

**Authors:** Marco Giuseppe Del Buono, Juan Ignacio Damonte, Cory R. Trankle, Dinesh Kadariya, Salvatore Carbone, Georgia Thomas, Jeremy Turlington, Roshanak Markley, Justin M. Canada, Giuseppe G. Biondi‐Zoccai, Michael C. Kontos, Benjamin W. Van Tassell, Antonio Abbate

**Affiliations:** 1 Division of Cardiology, Department of Internal Medicine, VCU Pauley Heart Center, Virginia Commonwealth University, West Hospital, West Wing 5-020, 1200 E Broad Street, P.O. Box 980204, Richmond, VA 23298 USA; 2 Department of Cardiovascular and Thoracic Sciences, Fondazione Policlinico Universitario A. Gemelli IRCCS, Catholic University of the Sacred Heart, Rome, Italy; 3 Interventional Cardiology Department, Hospital Italiano de Buenos Aires, Buenos Aires, Argentina; 4 Department of Kinesiology and Health Sciences, College of Humanities and Sciences, Virginia Commonwealth University, Richmond, VA USA; 5 Department of Medical-Surgical Sciences and Biotechnologies, Sapienza University of Rome, Latina, Italy; 6 Mediterranea Cardiocentro, Naples, Italy; 7 Department of Pharmacotherapy and Outcome Sciences, School of Pharmacy, Virginia Commonwealth University, Richmond, VA USA

**Keywords:** Cardiovascular biology, Clinical pharmacology, Molecular medicine

## Abstract

Leukocytosis is a common finding in patients with ST elevation myocardial infarction (STEMI) and portends a poor prognosis. Interleukin 1-β regulates leukopoiesis and pre-clinical studies suggest that anakinra (recombinant human interleukin-1 [IL-1] receptor antagonist) suppresses leukocytosis in myocardial infarction. However, the effect of IL-1 blockade with anakinra on leukocyte count in patients with STEMI is unknown. We reviewed the white blood cell (WBC) and differential count of 99 patients enrolled in a clinical trial of anakinra (n = 64) versus placebo (n = 35) for 14 days after STEMI. A complete blood cell count with differential count were obtained at admission, and after 72 h, 14 days and 3 months. After 72 h from treatment, anakinra compared to placebo led to a statistically significant greater percent reduction in total WBC count (− 35% [− 48 to − 24] vs. − 21% [− 34 to − 10], *P* = 0.008), absolute neutrophil count (− 48% [− 60 to − 22] vs. − 27% [− 46 to − 5], *P* = 0.004) and to an increase in absolute eosinophil count (+ 50% [0 to + 100] vs. 0% [− 50 to + 62], *P* = 0.022). These changes persisted while on treatment at 14 days and were no longer apparent at 3 months after treatment discontinuation. We found that in patients with STEMI IL-1 blockade with anakinra accelerates resolution of leukocytosis and neutrophilia. This modulation may represent one of the mechanisms by which IL-1 blockade improves clinical outcomes.

## Introduction

ST‐segment–elevation myocardial infarction (STEMI) is a leading cause of morbidity and mortality worldwide. Reperfusion strategies have revolutionized its treatment by reducing infarct size and improving survival^1^. However, an inflammatory response is triggered by acute ischemia and amplified by the reperfusion, which contributes to cause further injury despite being essential for tissue healing^2^. Elevated leukocyte count is a marker of inflammation and is associated with greater infarct size, reduced systolic function, and adverse clinical outcomes in patients with STEMI^3–6^. In response to ischemic injury, the number of neutrophils in the blood increases rapidly and begin to appear in the infarcted tissue within hours. While neutrophils contribute to the clearance of pathogens or debris as well as coordinate the monocyte-derived macrophage infiltration that activate reparative pathways necessary for scar formation^7–9^, perturbation of this finely regulated balance may lead to an exuberant and prolonged inflammatory state that acutely worsens the infarct size and chronically contributes to post-myocardial infarction adverse remodeling^10–13^.

Interleukin 1-β regulates leukopoiesis favoring a sustained neutrophil production in the bone marrow^8^. In this regard, pre-clinical studies reported that anakinra (recombinant human interleukin-1 [IL-1] receptor antagonist) suppressed leukocytosis in mouse models of myocardial infarction suggesting its potential role in suppressing the leukopoiesis in the bone marrow and neutrophil recruitment within the heart^14^.

Data on the role IL-1β in patients with STEMI are limited. In a small pilot trial of anakinra in 10 patients with STEMI, patients treated with anakinra had significantly lower leukocyte and neutrophil counts 24 h after the first injection compared to placebo-treated patients^15^, and leukocytes from anakinra-treated patients had reduced production of pro-inflammatory cytokines^16^. In this study we describe the effect of IL-1 blockade on leukocyte count in patients with STEMI treated with one of two different doses of anakinra in the setting of a randomized double-blinded clinical trial.

## Methods

### Study design

In the Virginia Commonwealth University Anakinra Remodeling Trial (VCUART) 3 (www.clinicaltrials.gov NCT01950299)^17,18^, 99 patients were randomly assigned to receive anakinra 100 mg once daily (standard dose), alternating with placebo every 12 h, for 14 days; anakinra, 100 mg twice daily, every 12 h (high dose) for 14 days; or placebo twice daily every 12 h for 14 days, with the first dose administered within 12 h of coronary angiography. Patients were excluded from the study if they had contraindications to treatment with anakinra, chronic inflammatory or infectious disease, or preexisting structural or functional severe cardiac abnormalities. For the purpose of this analysis, we pooled the two anakinra arms together, as previously presented for the clinical outcomes^17^.

### Laboratory data

The methods have been described in detail elsewhere^17,18^. A differential blood cell count was obtained at admission, and after 72 h, 14 days and 3 months. Total white blood cell (WBC) count and neutrophil, lymphocyte, monocyte and eosinophil counts were calculated using a hematology analyzer. Neutrophil to lymphocyte ratio, calculated as total neutrophil counts divided by total lymphocyte counts, was computed from the absolute values of neutrophils and lymphocyte^17,18^.

Moreover, to address whether changes in leukocyte counts may serve as biomarker for IL-1 blockade, we compared the changes in WBC count and absolute neutrophil count between baseline, 72 h, 14 days and 3 months in those patients who received anakinra and had heart failure clinical events (new-onset heart failure or death) and those who received anakinra and had heart failure clinical events at follow-up.

### Statistical analysis

The methods have been described in detail elsewhere^17,18^. Descriptive statistics were used to describe baseline and clinical characteristics of the patients. Categorical variables are presented as frequency (percentage) and compared using Chi-Square or Fisher's exact test as appropriate. Continuous variables are presented as median [interquartile range, IQR] and compared using Mann–Whitney U test or Spearman’s rank test for correlations**.** There were no differences in the missing data between the groups; no missing data imputation was used. All analyses were completed using SPSS, version 24.0 (SPSS; Chicago, IL) with significance set at α = 0.05^17,18^.

### Regulatory data

All methods were carried out in accordance with relevant guidelines and regulations. All experimental protocols were approved by the Virginia Commonwealth University Institutional Review Board. All subjects provided written informed consent to be part of the trial in accordance with the Virginia Commonwealth University Institutional Review Board.

## Results

### Baseline characteristics

Baseline characteristics of the 99 patients have been previously reported^17^ and are summarized in Table 1. Patients were predominantly male (n = 80, 81%) with a median age of 55 [49.0–62.0] years. Patients were randomized to anakinra standard dose daily (n = 33, [33%]), anakinra high dose (n = 31, [31%]), or placebo (n = 35, [35%]). Clinical characteristics were well matched, without statistically significant differences between anakinra and placebo groups except for a higher prevalence of diabetes mellitus in the placebo compared to anakinra group (15 [43%] vs. 15 [23%], *P* = 0.044). There were no differences between the two groups in time from symptom onset to percutaneous coronary intervention, time from symptom onset to investigational drug administration, or periprocedural drugs received. There was no difference in infarct size between the two groups, while the under the curve for high sensitivity C-Reactive Protein (CRP) was significantly lower in patients receiving anakinra versus placebo (67 [39–120] vs. 214 [131–394] mg·day/L, *P* < 0.001) (Table 2).

**Table 1:** Clinical characteristics of the patients in anakinra and placebo groups.

	Anakinra (n = 64)	Placebo (n = 35)	*P-*value
Age, y	55 [48–61]	56 [51–65]	0.174
Female sex	14 (22)	5 (4)	0.359
White	36 (56)	21 (60)	0.223
Black	21 (33)	6 (17)	
Hispanic	2 (3)	3 (9)	
Other	5 (8)	5 (14)	
**Procedural characteristics**			
Symptom onset to PCI, min	187 [106–333]	180 [130–347]	0.801
Symptom onset to investigational drug administration, min	508 [348–718]	529 [403–716]	0.669
Fibrinolytic use before PCI	5 (8)	3 (9)	0.587
PCI type			
Primary PCI	59 (92)	32 (91)	0.587
PCI after fibrinolysis	5 (8)	3 (9)	0.587
Use of drug-eluting-stent	44 (69)	30 (86)	0.063
Use of thrombectomy	10 (16)	6 (17)	0.844
Use of P2Y12 inhibitor	64 (100)	35 (100)	1
Clopidogrel	9 (14)	7 (20)	
Prasugrel	22 (34)	12 (34)	
Ticagrelor	33 (52)	15 (46)	
**Clinical characteristics**			
Coronary artery disease	14 (22)	7 (20)	0.827
Diabetes mellitus	15 (23)	15 (43)	**0.044**
Systemic arterial hypertension	33 (52)	23 (66)	0.174
Baseline LVEF, %	51 [44–58]	53 [42–57]	0.963

*STEMI* ST elevation myocardial infarction, *PCI* percutaneous coronary intervention, *Min* minutes, *LVEF* left ventricle ejection fraction.

Significant value are in [bold].

**Table 2:** Laboratory data according to anakinra and placebo.

	Anakinra (n = 64)	Placebo (n = 35)	*P*-value
CKMB-AUC, ng/mL*d	2219 [1130–3821]	2351 [765–4668]	0.859
**At admission**			
Hemoglobin, g/dL	14.5 [13.4–15.3]	14.4 [13.6–15.6]	0.692
Hematocrit, %	43 [40–47]	42 [41–44]	0.558
White blood cell, 10^9^/L	10.85 [8.52–13.90]	11.40 [9.20–15.07]	0.692
Absolute neutrophil count, 10^9^/L	7.45 [4.72–11.05]	7.30 [5.20–12.50]	0.622
Absolute lymphocyte count, 10^9^/L	2.05 [1.3–3.02]	1.8 [1.30–2.55]	0.699
Absolute monocyte count, 10^9^/L	0.60 [0.40–0.80]	0.70 [.50–0.80]	0.185
Absolute eosinophil count, 10^9^/L	0.10 [0.00–0.125]	0.10 [0.00–0.20]	0.279
Neutrophil to lymphocyte ratio	4.29 [1.99–7.51]	3.71 [2.08–8.37]	0.719
Creatinine, mg/dL	0.94 [0.78–1.10]	1.00 [0.89–1.34]	0.063
NTproBNP, pg/mL	52.50 [22.00–217.59]	95.5 [23.75–244.00]	0.614
**At 72 h**			
White blood cell, 10^9^/L	7.50 [6.20–8.35]	8.30 [7.20–9.80]	
% Change from baseline	− 35% [− 48 to − 24]	− 21% [− 34 to − 10]	**0.008**
Absolute neutrophil count, 10^9^/L	4.15 [3.22–5.07]	5.40 [4.65–5.40]	
% Change from baseline	− 48% [− 60 to − 22]	− 27% [− 46 to − 5]	**0.004**
Absolute lymphocyte count, 10^9^/L	2.15 [1.50–2.60]	1.80 [1.40–2.20]	
% Change from baseline	− 7% [− 4% to + 32]	− 6% [− 38 to + 22]	0.657
Absolute monocyte count, 10^9^/L	0.60 [0.50–0.80]	0.80 [0.60–0.95]	
% Change from baseline	+ 11% [− 29 to + 51]	+ 14% [− 13 to + 47]	0.604
Absolute eosinophil count, 10^9^/L	0.20 [0.10–0.30]	0.10 [0.10–0.30]	
% Change from baseline	+ 50% [0 to + 100]	0% [− 50 to + 62]	**0.022**
Neutrophil to lymphocyte ratio	1.90 [1.37–3.22]	3.35 [2.6	
% Change from baseline	− 54% [− 13 to + 70]	− 13% [− 58 to + 70]	**0.047**
**At 14 days**			
White blood cell, 10^9^/L	7.10 [5.70–9.40]	8.60 [6.92–10.5]	
% Change from baseline	− 33% [− 45 to − 22]	− 20% [− 41 to − 9]	**0.044**
Absolute neutrophil count, 10^9^/L	4.60 [3.30–5.50]	5.50 [4.00–7.15]	
% Change from baseline	− 42% [− 61 to − 25]	− 32% [− 51% to − 1]	0.067
Absolute lymphocyte count, 10^9^/L	2.10 [1.50–2.60]	2.00 [1.70–2.27]	
% Change from baseline	0% [− 32 to + 32]	+ 10% [− 22 to 40]	**0.313**
Absolute monocyte count, 10^9^/L	0.60 [0.50–0.80]	0.65 [0.50–0.70]	
% Change from baseline	− 5% [− 30 to + 26]	0% [− 20 to + 3]	0.989
Absolute eosinophil count, 10^9^/L	0.20 [0.10–0.30]	0.20 [0.20–0.30]	
% Change from baseline	+ 100% [0 to + 200]	0% [− 30 to + 100]	**0.043**
Neutrophil to lymphocyte ratio	2.26 [1.34–3.25]	2.89 [1.93–3.68]	
% Change from baseline	− 50% [− 67 to 10]	− 36% [− 65 to + 3]	0.500
**At 3 months**			
White blood cell, 10^9^/L	6.90 [5.90–8.60]	7.55 [6.05–9.47]	
% Change from baseline	− 41% [− 49 to − 16]	− 30% [− 44 to − 19]	0.301
Absolute neutrophil count, 10^9^/L	4.20 [3.30–5.20]	4.35 [3.50–5.70]	
% Change from baseline	− 53% [− 61 to − 25]	− 43% [− 54 to − 33]	0.298
Absolute lymphocyte count, 10^9^/L	2.10 [1.50–2.60]	2.00 [1.57–2.30]	
% Change from baseline	− 3% [− 29 to + 35]	+ 8% [− 28 to + 59]	0.584
Absolute monocyte count, 10^9^/L	0.60 [0.50–0.70]	0.65 [0.50–0.70]	
% Change from baseline	− 10% [− 28 to + 24]	7% [− 30 to + 25]	0.748
Absolute eosinophil count, 10^9^/L	0.20 [0.10–0.20]	0.20 [0.10–0.32]	
% Change from baseline	0% [0 to 100]	+ 33% [0–100%]	0.764
Neutrophil to lymphocyte ratio	2.09 [1.35–2.85]	2.61 [1.73–3.24]	
% Change from baseline	− 51% [− 70 to 17]	− 42% [− 75 to − 9]	0.964

Data are expressed as median [interquartile range]. *P*-values in bold character indicate significant values (< 0.05) for difference between groups.

*CKMB-AUC* Creatine kinase-MB area under the curve, *CRP-AUC* C-reactive protein area under the curve, *NTproBNP* N-terminal pro-brain natriuretic peptide.

### White blood cell count and differential count during STEMI

Table 2 shows WBC count with differential and other laboratory parameters in the two groups. There were no significant differences in any of the laboratory parameters at baseline. A significant reduction in WBC (11.40 [9.20–15.07] vs. 8.30 [7.20–9.80] 10^9^/L; − 21% [− 34 to − 10], *P* < 0.001 within placebo group) and neutrophil count (7.30 [5.20–12.50] vs. 5.40 [4.65–5.40] 10^9^/L; − 27% [− 46 to − 5], *P* = 0.003 within placebo group) were seen in the placebo group at 72 h (Table 2).

### Effect of treatment with anakinra on white blood count

**Figure 1:**
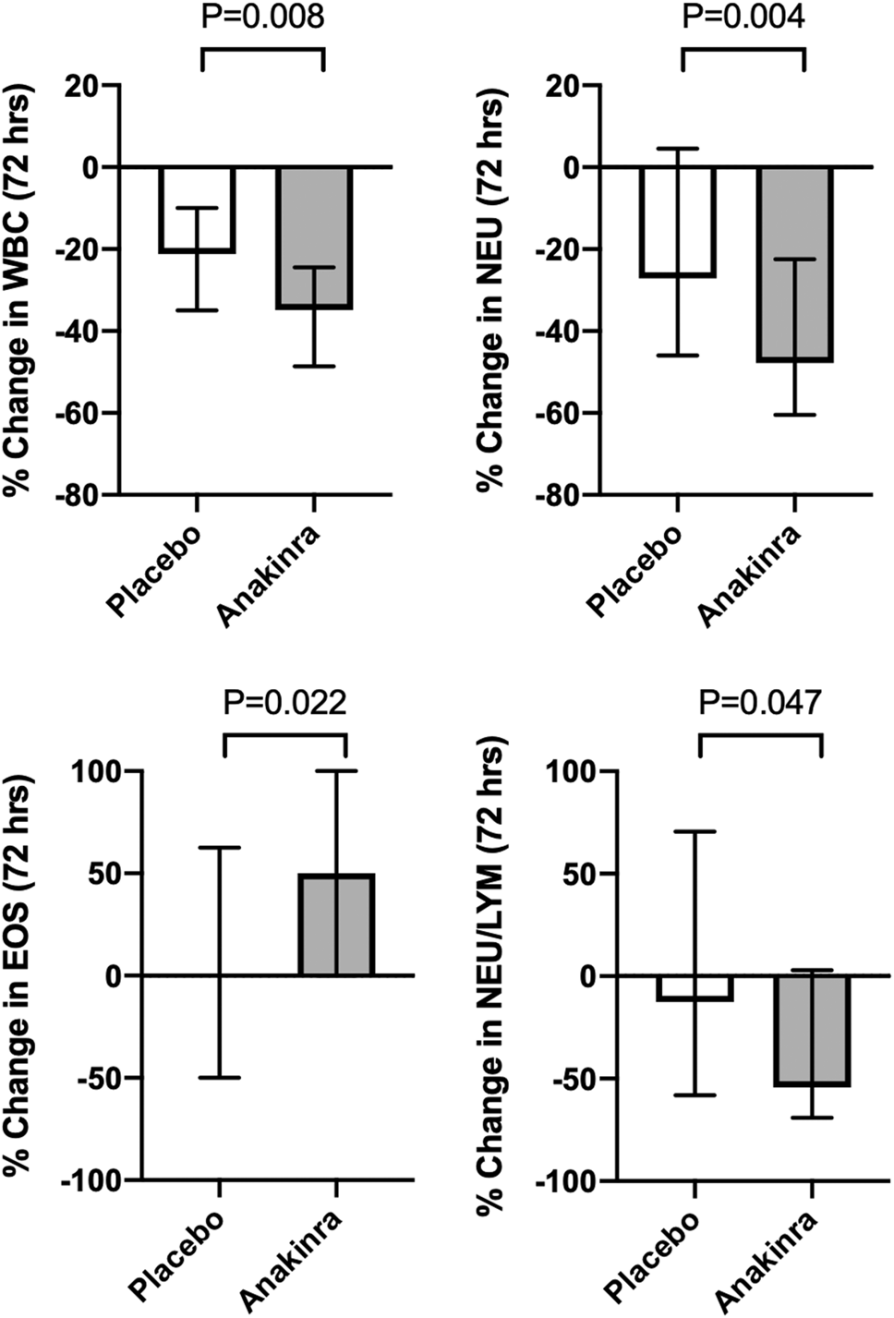
Percentage (%) change from baseline to 72 h in white blood cell count (WBC; panel **A**), absolute neutrophil count (NEU; panel **B**), absolute eosinophils count (EOS; panel **C**) and neutrophil to lymphocyte ratio (NEU/LYM; panel **D**) in the anakinra (n = 64) versus placebo group (n = 35).

At 72 h, when compared with placebo, treatment with anakinra led to a statistically significant greater percentage reduction in total WBC count (− 35% [− 48 to − 24] vs. -21% [− 34 to − 10], *P* < 0.001 within anakinra group, and *P* = 0.008 for between groups differences) (Fig. 1, Table 2). A significantly greater percentage reduction in WBC count persisted in the anakinra group compared to placebo group at 14 days, while on treatment, and it was no longer seen at 90 days following discontinuation of treatment (Fig. 1, Table 2).

### Effect of treatment with anakinra on leukocyte differential count

At 72 h, when compared with placebo, treatment with anakinra led to a statistically significant greater percentage reduction in absolute neutrophil count (− 48% [− 60 to − 22] vs. − 27% [− 46 to − 5], *P* < 0.001 within anakinra group, and *P* = 0.004 for between groups differences), and neutrophil to lymphocyte ratio (− 54% [− 13 to + 70] vs. − 13% [− 58 to + 70], *P* < 0.001 within anakinra group, and *P* = 0.047 for between groups differences), and to an increase in absolute eosinophil count (+ 50% [0 to + 100] vs. 0% [− 50 to + 62], *P* < 0.001 within anakinra group, and *P* = 0.022 for between groups differences)(Fig. 1, Table 2). A significantly greater percentage increase in absolute eosinophil count persisted in the anakinra group compared to placebo group at 14 days, while on treatment, and it was no longer seen at 90 days following discontinuation of treatment (Fig. 1, Table 2).

Patients who received anakinra and had heart failure related clinical events at follow-up had a trend towards a smaller percentage reduction in WBC (− 28% [− 31 to − 3] vs. − 38% [− 49 to − 26], *p* = 0.08) and a significantly smaller reduction in absolute neutrophil count (− 8% [− 23 to + 26] vs. − 53% [− 60 to − 30], *p* = 0.011) at 72 h compared to those receiving anakinra and had no heart failure related clinical events at follow-up. Patients who received anakinra and had adverse heart failure clinical events at follow-up had also a significantly smaller percentage reduction in WBC (− 19% [− 29 to + 5] vs. − 35% [− 46 to − 25] *p* = 0.013) and absolute neutrophil count (− 6% [− 40 to + 77] vs. − 48% [− 64 to − 29], *p* = 0.011) at 14 days compared to those receiving anakinra and had no adverse HF related events at follow-up. No significantly differences in changes in WBC and absolute neutrophil count between baseline and 3 months were observed.

## Discussion

We herein report, for the first time in literature, that IL-1 blockade with anakinra leads to a significantly greater reduction of leukocyte count in patients with STEMI, and that it drives a relative greater reduction in neutrophils and an increase in eosinophils. Considered a surrogate for the infiltration of WBC into necrotic tissue in response to ischemia and reperfusion, leukocytosis is a common finding in patients with acute STEMI and portends a poor prognosis^3–6,19–21^. Leukocytosis is considered a surrogate for the infiltration of WBC into necrotic tissue in response to ischemia and reperfusion. Neutrophils are the first cells to arrive in the infarcted tissue attracted by the cellular debris and damage-associated molecular patterns generated by the necrotic cells^7–9^. Upon arrival, leukocytes become activated and generate reactive oxygen species and proteolytic enzymes—thereby further expanding myocardial injury^7–9^. Recently, Sreejit and colleagues showed that neutrophils play a key role in determining the nature and orchestrating the inflammatory response in the heart^9^. Once recruited in the myocardium, activated neutrophils may release various proteins that prime the Nod-Like-Receptor (NLR) family Pyrin Domain-Containing 3 (NLRP3) inflammasome—a multi-molecular platform crucial to induction of the inflammatory response to cellular danger—on naïve neutrophils and stimulate them to produce IL-1β locally. This local production interacts with the IL-1 receptor type I (IL-1R_I_) on hematopoietic stem cells in the bone marrow, stimulating myelopoiesis in a cell-intrinsic manner and amplify the granulopoiesis^9^. Unopposed IL-1 activity during myocardial infarctions mobilizes myeloid cells from bone marrow to the infarction site inducing pathological myocardial healing and favoring cardiac rupture in experimental models^5,20^. During acute myocardial infarction, an increased expression of the IL-1R_I_ was found, suggesting that the upregulation of IL-1R_I_ may facilitate neutrophil proliferation and differentiation^9^. Furthermore, IL-1 stimulates the calcium-dependent degranulation of neutrophils and release of proteases (e.g. cathepsin G, elastase, and proteinase) that may contribute to the proteolytic break down of necrotic myocytes and extracellular matrix, and to cleave and activate multiple IL-1 family members^22^. Of note, IL-1 is sufficient to induce a cardiomyopathy phenotype in the mouse^23^.

From a translational point of view, in the VCUART phase II clinical trial program that included 3 studies (n = 139)^15,17,24,25^ of patients with STEMI treated with anakinra resulted in a significant improvement in left ventricular performance and a significant reduction of new-onset heart failure and of heart failure hospitalization versus placebo. Similarly, the CANTOS trial enrolling patients with previous acute myocardial infarction and persistent inflammation found that canakinumab (an IL-1β blocker) resulted in a reduction in the hospitalizations for heart failure^26^.

The larger reduction in WBC in patients treated with anakinra who were free of heart failure related events at follow up suggests that changes in leukocyte counts may serve as favorable prognostic biomarker for IL-1 blockade in STEMI (responders versus non-responders).

We also found that treatment with anakinra led to a significant increase in circulating eosinophils. Eosinophilia is reported in 9% of patients using anakinra in clinical practice^27^. The underlying mechanisms are not known. However, eosinophils have recently emerged to play an important role in infarct healing. Eosinophil recruitment within the myocardium may assist in mitigating the cardiac inflammatory cell profile, limiting cardiomyocyte apoptosis, modulating fibroblast activity, and regulating post myocardial infarction heart inflammatory cell adhesion and infiltration^28^. Whether the benefits of anakinra in modulating the inflammatory response and preventing post-STEMI heart failure is also related to an effect on eosinophils is unknown.

The small sample size of the study population and the post-hoc nature of the analysis represent the major limitations of this report. The white blood cell count with differential count is also an approximate measure of leukocyte populations with no insight in the subpopulations thus limiting the ability to fully understand the process. Furthermore, we did not measure values of WBC count and its differential count at 12 and 24 h, when a peak in leukocytes and neutrophils is expected during STEMI^3^, and we may be therefore unable to assess the effect of anakinra on leukocyte in the early phase during STEMI and to fully appraise the effects of anakinra.

In conclusion, IL-1 blockade with anakinra resulted in a greater reduction of leukocyte count in patients with STEMI, with a relative reduction in neutrophils and increase in eosinophils. These data support the pathophysiologic role of IL-1 in the leukopoiesis in acute myocardial infarction and support the role of therapeutic strategies aiming at reducing IL-1 signaling or inhibiting the upstream inflammasome to target inflammation and improve infarct healing and outcomes in STEMI.
